# Should Gallbladder Pathologies Be Investigated in Patients With Colon Polyps?

**DOI:** 10.7759/cureus.71545

**Published:** 2024-10-15

**Authors:** Nihan Turhan, Didem Ertorul, Cengiz Duran, Meryem Gözde Kılıç, Taha Yusuf Kuzan, Servan Yaşar, Dilek Yılmaz, Elbrus Zarbaliyev

**Affiliations:** 1 General Surgery, Sancaktepe Martyr Prof. Dr. İlhan Varank Training and Research Hospital, Istanbul, TUR; 2 Radiology, Sancaktepe Martyr Prof. Dr. İlhan Varank Training and Research Hospital, Istanbul, TUR; 3 Pathology, Sancaktepe Martyr Prof. Dr. İlhan Varank Training and Research Hospital, Istanbul, TUR; 4 General Surgery, Istanbul Yeni Yuzyıl University, Gaziosmanpasa Hospital, Istanbul, TUR

**Keywords:** colonoscopy, colon polyp, gallbladder polyp, gallbladder stone, ultrasonography

## Abstract

Background

Data regarding the frequency of development of gallbladder pathologies in patients with colon polyps are quite limited and heterogeneous. Hence, this study aimed to investigate whether colon polyps cause an increased risk of developing gallbladder pathologies and the necessity of ultrasonographic screening.

Methodology

This retrospective, cross-sectional study was conducted among adult patients who underwent a colonoscopy and hepatobiliary ultrasound as part of their health check-up from January 2018 to January 2023. The frequency, etiological factors, and association of gallbladder pathologies with colon polyps were investigated.

Results

A total of 128 patients were included in the study. When the distribution of colon polyp pathology was examined, 78.9% (n = 101) were adenomas, 18.8% (n = 24) were non-neoplastic polyps, and 2.3% (n = 3) were adenocarcinomas. The rate of patients with gallbladder stones was 10.9% (n = 14) and the rate of patients with polyps was 18.8% (n = 24). On multivariate logistic regression analysis, variables found to be significant for the development of gallbladder pathologies were determined as obesity, hyperlipidemia, and polyp’s distal colon location.

Conclusions

Obesity and metabolic syndrome were among the etiological factors in this patient group. Hepatobiliary ultrasound, a non-invasive and inexpensive examination, is useful in patients with colon polyps.

## Introduction

The high incidence of colon and rectal cancers worldwide has led to the inclusion of these diseases in screening programs for early diagnosis. The evolution of polyps detected in the colon from adenoma to carcinoma over time demonstrates the importance of early identification of these lesions [1-3]. The increasing detection of colon polyps in parallel with increased screening programs has raised the question of whether these lesions can be associated with other diseases [4,5].

Gallbladder pathologies are very common diseases in society. According to the literature, these numbers go up to 20% in adults for gallbladder stones and 6.9% for gallbladder polyps [2]. Embryologically, it has been shown that the gallbladder epithelium and the colon epithelium develop from the same epitope. When the etiology is investigated, gallbladder polyps and gallbladder stones share some risk factors with colorectal polyps, such as age, obesity, metabolic abnormalities, hyperlipidemia, and glucose intolerance [6,7].

Very few studies in the literature have investigated the relationship between colon polyps and gallbladder pathologies. For these reasons, the risk of coexistence of gallbladder pathologies and colon polyps is an important research topic, especially for emphasizing public health-protective methods [8,9]. In many countries, there are colonoscopic screening programs for early diagnosis of colon cancer after the age of 40-45 years. However, such screening programs are not available for the hepatobiliary system. We believe that this study will help guide the selection of patients who should be evaluated for gallbladder pathologies as a result of colonoscopic screening. This study aims to investigate the etiological factors and association of gallbladder pathologies with colon polyps.

## Materials and methods

This retrospective study included adult patients who underwent colonoscopy check-ups and abdominal ultrasonography at our hospital between January 2018 and January 2023. The inclusion criteria included patients over 18 years old with colon polyps who had undergone hepatobiliary ultrasonography. Inadequate bowel preparation, incomplete colonoscopy, a history of familial adenomatous polyposis coli, inflammatory bowel disease, previous colon surgery, cholecystectomy, and patients with missing data were excluded from the study.

Age, gender, body mass index (BMI), family history of colorectal cancers, metabolic laboratory markers (Hemoglobin A1c (HbA1c), low-density lipoprotein (LDL)), and colonoscopic and ultrasonographic findings were recorded. BMI was defined as body weight (kg) divided by the square of body height (m^2^). Patients with a BMI over 30 kg/m^2^ were considered obese. A family history of colorectal cancer was determined to be positive if a first- or second-degree relative had a history of colon cancer.

The colonoscopy procedures were performed by experienced surgeons. The size, location, and number of colonic lesions were recorded. The colorectal polyps were recorded as being in the proximal colon (proximal to splenic flexure) or distal colon (distal to splenic flexure). The polyps were examined histopathologically based on the World Health Organization classification. They were grouped into three non-neoplastic polyps (hyperplastic polyps, hamartomas, lymphoid aggregates, and non-specific colitis), colorectal adenomas (low-grade adenomas, tubular adenomas, adenomatous polyps, and villous histology with high-grade dysplasia), and adenocarcinoma.

Ultrasonography of the patients was performed by experienced radiologists. The presence of gallbladder stones, gallbladder polyps, and hepatosteatosis was recorded. Gallbladder stones were detected as echogenic movable structures with acoustic shadowing in the gallbladder lumen. Gallbladder polyps were diagnosed as immobile, hyperechoic masses attached to the gallbladder wall with no acoustic shadowing.

The Kolmogorov-Smirnov test was used to assess whether the variables followed a normal distribution. Continuous variables were presented as median (range, minimum to maximum). Categorical variables were reported as numbers and percentages. According to the normality test results, the Mann-Whitney U test was used to compare the two groups. The Pearson chi-square test, Fisher’s exact test, and Fisher-Freeman-Halton test were used for comparing categorical variables. Multiple logistic regression analysis was performed to determine the risk factors affecting the incidence of gallbladder diseases. Variables were included in the multiple logistic regression model by using the forward likelihood ratio method. The significant variables in the model were independent variables. The multiple logistic regression models were accepted as statistically significant when the p-value was less than 0.001. SPSS version 21.0 (IBM Corp., Armonk, NY, USA)) was used for statistical analysis, and a p-value <0.05 was set as the threshold for statistical significance.

This study was approved by the Ethics Committee of Sancaktepe Martyr Prof. Dr. İlhan Varank Training and Research Hospital (approval number: 2023/59).

## Results

A total of 1,117 subjects underwent a total colonoscopy between January 2018 and January 2023. There were 169 patients with colon polyps. In total, 128 patients whose data could be accessed and who met the evaluation criteria were included in the study. The median age of the patients included in the study was 59 years (minimum = 27, maximum = 77). Baseline characteristics of the patients are presented in Table 1.

**Table 1:** Baseline characteristics of patients. Data are expressed as n (%).

Parameters		N = 128
Gender	Female	54 (42.2%)
	Male	74 (57.8%)
Obesity	No	75 (58.6%)
	Yes	53 (41.4%)
Hyperlipidemia	No	75 (58.6%)
	Yes	53 (41.4%)
Diabetes mellitus	No	69 (53.9%)
	Yes	59 (46.1%)
Hypertension	No	87 (68%)
	Yes	41 (32%)
Coronary artery disease	No	107 (83.6%)
	Yes	21 (16.4%)
Familial colorectal cancer history	No	114 (89.1%)
	Yes	14 (10.9%)

Colon polyps were more frequently (64.1%) distally located, and when pathology distributions were examined, the most frequently detected pathology was adenoma (78.9%). The rate of gallbladder stones was 10.9%, and the rate of gallbladder polyps was 18.8% (Table 2).

**Table 2:** Baseline characteristics of colon polyps and ultrasonographic findings. Data are expressed as n (%).

Parameters		N = 128
Colon polyp location	Distal	82 (64.1%)
	Proximal	46 (35.9%)
Colon polyp pathology	Adenoma	101 (78.9%)
	Non-neoplastic polyps	24 (18.8%)
	Adenocarcinoma	3 (2.3%)
Hepatosteatosis	No	73 (57%)
	Yes	55 (43%)
Gallbladder stone	No	114 (89.1%)
	Yes	14 (10.9%)
Gallbladder polyp	No	104 (81.3%)
	Yes	24 (18.8%)

In the univariate analyses, in patients with colon polyps, the frequency of gallbladder pathologies was statistically higher in obese, hyperlipidemic, and diabetic patients (p = 0.009, p = 0.027, and p = 0.020, respectively) (Table 3).

**Table 3:** Univariate analyses for gallbladder diseases. Data are expressed as n (%), median (minimum-maximum), and mean ± standard deviation. ^a^: Mann-Whitney U test; ^b^: Pearson chi-square test; ^c^: independent sample t-test; ^d^: Fisher’s exact test.

Parameters		Gallbladder disease (stone and/or polyp)		
		Present (n = 35)	Absent (n = 93)	P-value
Age		61 (36–76)	58 (27–77)	0.383^a^
Gender	Female	16 (45.7%)	38 (40.9%)	0.620^b^
	Male	19 (54.3%)	55 (59.1%)	
Obesity		21 (60%)	32 (34.4%)	0.009^b^
Hyperlipidemia		20 (57.1%)	33 (35.5%)	0.027^b^
Diabetes mellitus		22 (62.9%)	37 (39.8%)	0.020^b^
Hypertension		15 (42.9%)	26 (28%)	0.107^b^
Coronary artery disease		8 (22.9%)	13 (14%)	0.227^b^
Hemoglobin A1c level		6 (5.10–11)	5.70 (4.80–11.50)	0.149^a^
Low-density lipoprotein level		125.26 ± 33.32	114.76 ± 35.12	0.129^c^
Familial colorectal carcinoma history		6 (17.1%)	8 (8.6%)	0.205^d^

The frequency of gallbladder pathologies was higher in the distally located colon polyp group (p = 0.007) (Table 4).

**Table 4:** Gallbladder disease presence according to colon polyp features. Data are expressed as n (%). ^b^: Pearson chi-square test; ^e^: Fisher-Freeman-Halton test.

Parameters		Gallbladder disease (stone and/or polyp)		
		Present (n = 35)	Absent (n = 93)	P-value
Colon polyp location	Distal	29 (82.9%)	53 (57%)	0.007^b^
	Proximal	6 (17.1%)	40 (43%)	
Colon polyp pathology	Adenoma	28 (80%)	73 (78.5%)	>0.99^e^
	Non-neoplastic polyps	6 (17.1%)	18 (19.4%)	
	Adenocarcinoma	1 (2.9%)	2 (2.2%)	

Patient’s age, gender, BMI, hyperlipidemia, diabetes mellitus, hypertension, Hba1c, LDL, coronary artery disease, family history of colorectal disease, colon polyp’s location, colon polyp’s pathology, and presence of hepatosteatosis in ultrasonography were included in the multivariate logistic regression analysis. In the multivariate logistic regression analysis, the variable selection process was performed using the forward elimination method, and in the final step, the variables found to be significant in the model were determined as BMI, hyperlipidemia, and colon location. The analysis results are presented in Table 5.

**Table 5:** Risk factors affecting the formation of gallbladder disease (stones and/or polyps) as a result of multivariate analysis. Model χ^2^ = 18.70; p < 0.001; Hosmer and Lemeshow test: p = 0.756. The “no obesity” category was accepted as the reference category for the BMI variable, the “none” category for the hyperlipidemia variable, and the “proximal” category for the colon location variable. OR: odds ratio; CI: confidence interval

Parameters	Wald	P-value	OR	95% CI	
				Minimum	Maximum
Obesity (yes)	5.76	0.016	2.809	1.208	6.531
Hyperlipidemia (yes)	4.76	0.029	2.567	1.101	5.988
Colon location (distal)	6.47	0.011	3.757	1.355	10.414

## Discussion

Only a few studies in the literature have evaluated the relationship between colon polyps and gallbladder pathologies [4,6,10]. Similar to previous studies, our study showed that obesity, diabetes, and hyperlipidemia were effective in the development of gallbladder pathologies in patients with colon polyps [11,12]. Geng et al. showed that gallbladder polyps were associated with colorectal adenomas in a retrospective cohort study of 1,662 patients. Similar to our study, they showed that obesity and metabolic syndrome were common etiological factors for the development of gallbladder polyps and colorectal adenomas. They also pointed out the bile acid mechanism in the development of gallbladder polyps and colorectal polyps. Gallbladder polyps are associated with higher concentrations of secondary bile acids. Similarly, fecal bile acids, lithocholic acid, and total secondary bile acid levels were higher in patients with adenomatous polyps [4,13].

In our study, gallbladder pathologies were more common in patients with polyps located in the distal colon. Geng et al. also showed a positive relationship between the development of gallbladder polyps and left colon polyps. They suggested that this may be due to the relatively longer stool retention in the left colon, which, in turn, may be related to the longer exposure of epithelial cells to bile acids and increased production of cytotoxic secondary bile acids in the left colon [4]. On the other hand, Lee et al. suggested that the rates of gallbladder polyps and stones were higher in patients with proximal colon polyps [14].

In our study, no difference was found between the genders in terms of the development of gallbladder pathology. While the study conducted by Liu et al. showed that men were at a higher risk for the development of gallbladder pathology in patients with gastrointestinal polyps, the study conducted by Geng et al. showed that women were at a higher risk [4,6].

Although gallbladder polyps are relatively rare, they are generally found in approximately 3-13.8% of the adult population. However, the frequency of gallbladder polyps in our study was 18.8% [15-18]. It is noteworthy that gallbladder polyps were more common in our study than in the general population. We did not find similar results in our literature review and we think that this should be a separate research topic.

Stergios et al. reported in their systematic review that gallbladder polyps can adequately predict future risk for colorectal neoplasia [8]. Geng et al. reported that gallbladder epithelium and colorectal mucosal epithelium share a common epitope [4]. Here, the need for colonoscopy screening in patients with gallbladder polyps is discussed considering the risk of future colorectal neoplasia.

Lee et al. demonstrated that the risk of colorectal neoplasia increases with worsening severity of fatty liver in the presence of gallbladder polyps [19]. In our study, hepatosteatosis was not shown to increase the risk of gallbladder pathology.

Our study has a few limitations. As the number of patients was limited, gallbladder pathologies including stones and polyps were evaluated in a single group. To our knowledge, this is the first study to report on this subject in our country. With the increase in screening programs, multicenter cohort studies with a larger number of patients should be planned to evaluate each gallbladder pathology.

## Conclusions

Colonic polyps and gallbladder pathologies are seen to present together due to similar etiological factors. Adding hepatobiliary ultrasound to the examination screening program of patients with colon polyps might be beneficial for the early detection of gallbladder pathologies which might pose risks to patient health.

## References

[1] Leslie A, Carey FA, Pratt NR, Steele RJ (2002). The colorectal adenoma-carcinoma sequence. Br J Surg.

[2] Citarda F, Tomaselli G, Capocaccia R, Barcherini S, Crespi M, Group TI (2001). Efficacy in standard clinical practice of colonoscopic polypectomy in reducing colorectal cancer incidence. Gut.

[3] Aksoy S, Yılmazlar A, Erçelik M (2023). Frequency and clinical impact of microsatellite instability in colorectal dysplasia subgroups. Turkish J Colorectal Dis.

[4] Geng W, Qin X, Yang P, Wang J, Yu J, Wang X (2022). Association of gallbladder diseases with risk of gastrointestinal polyps. BMC Gastroenterol.

[5] Jeun JW, Cha JM, Lee JI, Joo KR, Shin HP, Lim JU (2014). Association of gallbladder polyp with the risk of colorectal adenoma. Intest Res.

[6] Liu YL, Wu JS, Yang YC, Lu FH, Lee CT, Lin WJ, Chang CJ (2018). Gallbladder stones and gallbladder polyps associated with increased risk of colorectal adenoma in men. J Gastroenterol Hepatol.

[7] Ben Q, An W, Jiang Y (2012). Body mass index increases risk for colorectal adenomas based on meta-analysis. Gastroenterology.

[8] Stergios K, Damaskos C, Frountzas M, Nikiteas N, Lalude O (2016). Can gallbladder polyps predict colorectal adenoma or even neoplasia? A systematic review. Int J Surg.

[9] Szpakowski JL, Tucker LY (2020). Outcomes of gallbladder polyps and their association with gallbladder cancer in a 20-year cohort. JAMA Netw Open.

[10] Hong SN, Lee TY, Yun SC (2015). The risk of colorectal neoplasia in patients with gallbladder diseases. J Korean Med Sci.

[11] Kim JH, Lim YJ, Kim YH (2007). Is metabolic syndrome a risk factor for colorectal adenoma?. Cancer Epidemiol Biomarkers Prev.

[12] Ortiz AP, Thompson CL, Chak A, Berger NA, Li L (2012). Insulin resistance, central obesity, and risk of colorectal adenomas. Cancer.

[13] Bayerdörffer E, Mannes GA, Richter WO, Ochsenkühn T, Wiebecke B, Köpcke W, Paumgartner G (1993). Increased serum deoxycholic acid levels in men with colorectal adenomas. Gastroenterology.

[14] Lee KC, Jeng WJ, Hsu CM, Kuo CJ, Su MY, Chiu CT (2019). Gallbladder polyps are associated with proximal colon polyps. Gastroenterol Res Pract.

[15] Kamaya A, Fung C, Szpakowski JL (2022). Management of incidentally detected gallbladder polyps: Society of Radiologists in Ultrasound Consensus Conference Recommendations. Radiology.

[16] Özkan C, Yılmaz S, Bozdağ E, Sıbıç O, Somuncu E (2024). Helicobacter pylori might be a contributing factor in gallbladder polyps or gallstones: a single center case-control matching study of Turkish individuals. Acıbadem Üniversitesi Sağlık Bilimleri Dergisi.

[17] Tian F, Ma YX, Liu YF, Liu W, Hong T, He XD, Qu Q (2022). Management strategy for gallbladder polypoid lesions: results of a 5-year single-center cohort study. Dig Surg.

[18] Metman MJ, Olthof PB, van der Wal JB, van Gulik TM, Roos D, Dekker JW (2020). Clinical relevance of gallbladder polyps; is cholecystectomy always necessary?. HPB (Oxford).

[19] Lee T, Yun KE, Chang Y, Ryu S, Park DI, Choi K, Jung YS (2016). Risk of colorectal neoplasia according to fatty liver severity and presence of gall bladder polyps. Dig Dis Sci.

